# ‘We keep her status to ourselves’: Experiences of stigma and discrimination among HIV-discordant couples in South Africa, Tanzania and Ukraine

**DOI:** 10.1080/17290376.2015.1014403

**Published:** 2015-03-17

**Authors:** Laetitia C. Rispel, Allanise Cloete, Carol A. Metcalf

**Affiliations:** ^a^ PhD, is a professor at the Centre for Health Policy & Medical Research Council Health Policy Research Group, School of Public Health, Faculty of Health Sciences, University of the Witwatersrand, Johannesburg, South Africa; ^b^ MA (Anthropology), is a researcher in the HIV/AIDS, Tuberculosis and Sexually Transmitted Infections (HAST) Research Programme, Human Sciences Research Council, Cape Town, South Africa; ^c^ MBBCh, MPH, is a doctor and epidemiologist at Médecins Sans Frontières, Cape Town, South Africa

**Keywords:** HIV-discordance, stigma, discrimination, couples, South Africa, Tanzania, Ukraine, sérodiscordants pour le VIH, stigmatisation, discrimination, couples, Afrique du Sud, Tanzanie, Ukraine

## Abstract

In HIV-discordant relationships, the HIV-negative partner also carries the burden of a stigmatised disease. For this reason, couples often hide their HIV-discordant status from family, friends and community members. This perpetuates the silence around HIV-discordant relationships and impacts on targeted HIV prevention, treatment and counselling efforts. This article reports on experiences of stigma and discrimination among HIV-discordant couples in South Africa, Tanzania and Ukraine. During 2008, HIV-discordant couples who had been in a relationship for at least one year were recruited purposively through health-care providers and civil society organisations in the three countries. Participants completed a brief self-administered questionnaire, while semi-structured interviews were conducted with each partner separately and with both partners together. Interviews were analysed using thematic content analysis. Fifty-one couples were recruited: 26 from South Africa, 10 from Tanzania, and 15 from Ukraine. Although most participants had disclosed their HIV status to someone other than their partner, few were living openly with HIV discordance. Experiences of stigma were common and included being subjected to gossip, rumours and name-calling, and HIV-negative partners being labelled as HIV-positive. Perpetrators of discrimination included family members and health workers. Stigma and discrimination present unique and complex challenges to couples in HIV sero-discordant relationships in these three diverse countries. Addressing stigmatisation of HIV-discordant couples requires a holistic human rights approach and specific programme efforts to address discrimination in the health system.

## Résumé

Dans les couples sérodiscordants pour le VIH, le partenaire séronégatif porte aussi le fardeau de la stigmatisation lié à la maladie. C'est pourquoi les couples sérodiscordants cachent souvent leur état sérologique à leur famille, à leurs amis et à la communauté. Cela perpétue le silence qui entoure les relations sérodiscordantes et a un impact important sur les efforts de prévention et de traitement et sur les conseils psychologiques en rapport avec le VIH. Dans cet article, il est question de la stigmatisation et de la discrimination vécues par les couples sérodiscordants en Afrique du Sud, en Tanzanie et en Ukraine. Au cours de l'année 2008, des couples sérodiscordants qui étaient dans une relation depuis au moins un an ont été intentionnellement recrutés par l'entremise de dispensateurs de soins de santé et d'organisations de la société civile dans les trois pays mentionnés. Les participants à l’étude ont répondu à un bref questionnaire autoadministré. Des entrevues semi-structurées ont été menées avec les partenaires, ensemble et séparément, et leur contenu a fait l'objet d'une analyse thématique. Cinquante-et-un couples ont été recrutés, dont 26 en Afrique du Sud, 10 en Tanzanie et 15 en Ukraine. La plupart des participants avaient divulgué leur séropositivité à quelqu'un d'autre que leur partenaire, mais peu de couples vivaient ouvertement leur sérodiscordance. Les expériences de stigmatisation étaient fréquentes et comprenaient les potins, les rumeurs, les injures et le fait pour le partenaire séronégatif d’être considéré d'emblée comme étant séropositif. La discrimination vécue provenait aussi des membres de la famille et des dispensateurs de soins de santé. La stigmatisation et la discrimination présentent des enjeux uniques et complexes pour les couples sérodiscordants dans les trois pays. Pour répondre à ces enjeux, il faut une approche globale basée sur les droits de la personne ainsi que des programmes ciblant spécifiquement la discrimination dans le système de santé.

## Introduction

The HIV pandemic continues to be a major global public health priority (UNAIDS & WHO 2013). South Africa, Tanzania and Ukraine, the three study countries, differ in their HIV epidemiological patterns and modes of transmission (UNAIDS & WHO 2013). Both South Africa and Tanzania experience generalised epidemics with primarily heterosexual transmission (UNAIDS & WHO 2013). HIV prevalence among persons aged 15–49 years is estimated to be 17.9% in South Africa and 5.1% in Tanzania (UNAIDS & WHO 2013). In contrast, Ukraine experiences a concentrated epidemic with transmission mainly occurring through injecting drug use, and it has an estimated HIV prevalence of 0.9% (UNAIDS & WHO 2013).

Studies have found that in sub-Saharan Africa 10–25% of new HIV infections occur in HIV-discordant couples (Chemaitelly, Shelton, Hallett & Abu-Raddad 2013; Dunkle, Stephenson, Karita, Chomba, Kayitesi, Vwalika, *et al*. 2008). The prevalence of HIV discordance among married and cohabitating couples in sub-Saharan Africa ranges from 3% to 20% in the general population, and 20% to 35% in couples in which one partner seeks care for HIV-related conditions (Chemaitelly *et al*. 2013; De Walque 2006; Guthrie, de Bruyn & Farquhar 2007; Lingappa, Lambdin, Bukusi, Ngure, Kavuma, Inamboa, *et al.* 2008). HIV sero-discordance therefore represents a neglected HIV prevention need (Kairania, Gray, Kiwanuka, Makumbi, Sewankambo, Serwadda, *et al*. 2010; UNAIDS & WHO 2013).

Research on HIV-discordance has been dominated by biomedical studies on the epidemiology of discordance and factors related to HIV transmission (Freeman & Glynn 2004; Guthrie *et al*. 2007; Hugonnet, Mosha, Todd, Mugeye, Klokke & Ndeki 2002; Lingappa *et al*. 2008). In recent years, there has been an increased focus on the psychosocial aspects of HIV-discordance (Persson 2008; Persson & Richards 2008; Rispel, Cloete, Metcalf, Moody & Caswell 2012; Rispel, Metcalf, Moody, Cloete & Caswell 2011). Notwithstanding the increased focus on the psychosocial aspects of HIV-discordance, studies have tended to focus on the reproductive decisions of sero-discordant couples (Cook, Hayden, Weiss & Jones 2014; Cooper, Harries, Myer, Orner & Bracken 2007; Cooper, Moodley, Zweigenthal, Bekker, Shah & Myer 2009; Rispel *et al*. 2011; Withers, Dworkin, Harrington, Kwena, Onono, Bukusi, *et al*. 2013). There is a dearth of studies on stigma experienced by couples in a sero-discordant relationship. Addressing stigma as part of the global HIV response remains a priority for national (SANAC 2011) and international (UNAIDS & WHO 2013) funding, policy development and programmes.

The departure point for much of the scholarly work on stigma remains Goffman's classic study (Goffman 1963), leading to a subsequent proliferation of research on conceptual refinements of stigma, the nature, sources and consequences of stigma, and demonstrations of the impact of stigma on the lives of the stigmatised (Brickley, Le Dung Hanh, Nguyet, Mandel, Giang le & Sohn 2009; Cloete, Simbayi, Kalichman, Strebel & Henda 2008; Earnshaw & Chaudoir 2009; Frye, Fortin, Mackenzie, Purcell, Edwards, Mitchell, *et al*. 2009). Although dynamic and multi-dimensional, often occurring within the context of unequal power relationships, different types of stigma have been identified, such as: internalised stigma; perceived stigma or the subjective awareness or fear of stigma; and enacted stigma or overt discrimination (Mall, Middelkoop, Mark, Wood & Bekker 2013; Parker & Aggleton 2003; Steward, Herek, Ramakrishna, Bharat, Chandy, Wrubel, *et al*. 2008; Thomas 2006; Weiss, Ramakrishna & Somma 2006).

In 2007, the Global Network of People Living with HIV (GNP+) commissioned an exploratory study on the psychosocial aspects of HIV discordance to inform their advocacy programmes. This paper discusses experiences of stigma and discrimination among 51 HIV sero-discordant couples in South Africa, Tanzania and Ukraine, who were recruited through health-care providers and civil society organisations and who had been in a relationship for at least one year.

## Methodology

In this study, HIV stigma refers to the ‘socially shared knowledge about the devalued status of people living with HIV (PLHIV), manifested in prejudice, discounting, discrediting, and discrimination directed at people perceived to have HIV and the individuals, groups, and communities with which they are associated’ (Steward *et al*. 2008: 1226).

The study was conducted in South Africa (Johannesburg and Cape Town), Tanzania (Dar es Salaam) and Ukraine (Kiev, Rivne and Ivano-Frankovsk) during 2008. The choice of study countries was determined largely by practical considerations, namely the location of the researchers; a convenient means of recruiting sero-discordant couples within a short time period and access to an appropriate in-country research ethics committee to provide ethics approval for the research. The population of interest was couples in long-term sexual relationships, in which one partner was HIV-positive, and the other HIV-negative. To be eligible to participate, the HIV-discordant couples were required to be in a relationship for at least one year, with known HIV-discordant status for at least 1 year previously, and both partners were required to be 18 years of age or older. Participants were not required to be legally married, to be living with their partner or to be monogamous. No testing or documentation was used to confirm the HIV status of either partner.

The couples were recruited purposively through health-care providers and civil society organisations in the three countries. In each country, ethics approval was obtained from an appropriate local ethics committee. Potential participants were approached by either a health-care provider or a person who was already aware of the couple's discordant status, in order to protect the privacy and respect the confidentiality of the individuals' HIV status. Researchers contacted potential participants who had given permission to be contacted. Individual written voluntary informed consent was obtained from each partner. All couples approached agreed to be interviewed in Tanzania and Ukraine. However, in South Africa, an additional nine sero-discordant couples were identified but were not included in the study, either because they could not be contacted or because one or both partners refused to participate.

Consent forms and measuring instruments were developed in English and translated into the predominant local languages. Participants were interviewed by trained fieldworkers in the language of their choice in their home or at a suitable, convenient venue. They completed a brief structured, self-administered questionnaire and participated in a semi-structured qualitative individual interview and a semi-structured couple interview. The self-administered questionnaire focused on demographic characteristics, history and duration of the current relationship, HIV-testing and health history of each partner, and involvement in HIV-related activities. The topics in the individual and couple interviews included: individual and couples' experiences of being in a discordant relationship; experiences of stigma and discrimination, including social pressure faced by both partners to have only HIV-concordant relationships; and disclosure of HIV status and issues related to family members and friends from the perspective of both the HIV-positive and HIV-negative partners. Participants were asked about their experiences of stigma and whether they had ever been discriminated against because of their or their current partners' HIV status. This approach recognises that social relations are lived and experienced through emotions (Parker & Aggleton 2003; Thomas 2006; VanDevanter, Stuart Thacker, Bass & Arnold 1999). Each set of interviews took between two and three hours. A voucher, equivalent in value to US$15, was given to each couple at the end of the interviews to thank them for their participation.

The self-administered questionnaires were coded, using a standard coding sheet and analysed using STATA^®^ 10. In South Africa and Tanzania, information on stigma, discrimination and disclosure from the individual and couple qualitative interviews was extracted, coded and included in the quantitative analysis. The qualitative interviews from South Africa and Tanzania were translated into English, transcribed and analysed using thematic content analysis (Miles, Huberman & Saldaña 2014). The steps consisted of: open coding using the participants' own words and phrases and without preconceived notions or classification; examining language used by each partner or couple; categorising the information from all the interviews and finally theoretical coding in which open codes and categories were compared to generate an analytic schema and to interpret the findings (Miles *et al*. 2014). Only a summary of the qualitative interviews from Ukraine was available due to logistical difficulties with translating the interviews back into English.

## Findings

Fifty-one couples were recruited: 26 from South Africa, 10 from Tanzania and 15 from Ukraine. The mean age of all participants was 34 years, with a range of 20–54. Fifty-three per cent of the HIV-positive participants were women. Couples had been in their current relationship for a mean period of six years. The vast majority of the couples were in heterosexual relationships, with only three homosexual couples, all of whom were located in South Africa. The Ukrainian participants were slightly younger, with a mean age of 29 years (range 20–39), compared with a mean age of 35 years among the South African participants and 37 years among the Tanzanian participants (Rispel, Metcalf, Moody & Cloete 2009). Seventy-three per cent (74/102) of participants were in employment, with 67% of HIV-positive participants in employment, compared with 78% of HIV-negative individuals (Rispel *et al*. 2009). The nature of these individuals' employment varied greatly, from low-skilled occupations such as cleaning and driving, to highly skilled occupations such as senior civil servants, managers and lawyers. The majority of couples (83%) lived together (South Africa: 19/26, 73%; Tanzania: 10/10, 100%; Ukraine: 13/15, 87%), and 58% had formalised their relationship either through marriage or a civil union (South Africa: 14/26, 54%; Tanzania: 5/10, 50%; Ukraine: 11/15, 73%) (Rispel *et al*. 2009).

The majority of participants in Tanzania (60%) had experienced overt discrimination, compared with 21% in South Africa. Forty-three per cent of the HIV-positive participants had experienced discrimination compared with 24% of the HIV-negative participants.

Couples' experiences of stigma included: dealing with gossip, rumours and name-calling, and ‘labelling’ (i.e. the assumption by others that the HIV-negative partner is HIV-positive); discrimination from family members and friends (including pressure to leave the discordant relationship); discrimination by health-care professionals; and broader community and societal discrimination. These categories overlap but are discussed separately for the sake of clarity.

### Gossip, rumours, name-calling and labelling

Participants reported stigmatising experiences of varying severity, as illustrated by the following quotes:

[Discrimination occurs] in a more subtle way. For example there are rumours that my partner wants attention, and that's why he is having a relationship with an HIV-positive woman. My partner got calls from his ex-girlfriends who are questioning his choices. (HIV-positive woman, Couple 4, South Africa)

I quarrelled with one lady at work, who then discussed me with other colleagues … that I am sick with HIV … (HIV-positive woman, Couple 15, South Africa)

Family and community members have limited knowledge or understanding of HIV discordance and they also have to deal with a ‘positive by association’ perception following disclosure:

I told my family that I am living in [a] discordant relationship, even though relatives did not believe that I am [HIV-] negative, while my partner is [HIV-] positive. My friends are making me scared of staying with a positive partner. They are asking questions every day. (HIV-negative woman, Couple 3, Tanzania)

I experienced shock and disbelief from family and friends. Most could not understand the fact that I am [HIV-] negative and my wife is HIV-positive. (HIV-negative man, Couple 14, South Africa)

### Discrimination from family members and friends

In those instances where couples had disclosed to family members and friends, they reported experiences of stigma and discrimination. Couples reported both subtle and overt pressure from families, especially those of the HIV-negative partners, and friends, to leave the discordant relationship.

I fought a battle with my family, I explained to my parents. My parents are protective, and they are worried that we will default … so they keep a watchful eye. We still battle to be together. (HIV-negative man, Couple 4, South Africa)

The family labels us and calls us stigmatising names. My wife's relatives are the most stigmatising especially during festivals like marriage … We are given names like ‘walking corpse’. (HIV-negative man, Couple 7, Tanzania)

My mother once said that she knows about our misfortune [wife is HIV-positive] but we never talked about it after that. My brother probably guesses too, but we don't discuss these issues with him either. (HIV-negative man, Ukraine)

Couples also have to deal with the perceptions of or pressure from friends who have been taken into confidence about the couples' HIV-discordant status.

Yes, I have experienced discrimination … sometimes friends are saying: Why are you living with an HIV-positive woman? You are still young, you can get another woman. (HIV-negative man, Couple 1, Tanzania)

I disclosed to my best friend, and my mom. My brother and sister were totally against the relationship. My friend wanted to find out if I am certain of starting a relationship with a partner who is HIV-positive and if I understand the implications and consequences of being in such a relationship. (HIV-negative woman, Couple 17, South Africa)

### Discrimination by health-care professionals

In Ukraine, discrimination by health-care professionals was a reported problem.

I needed serious surgery on my jaw. When I applied to medical professionals, the surgeons, informing them about my positive status, I was refused on the basis of all kinds of made-up reasons. (HIV-positive man, Rivne, Ukraine)

When I need medical assistance I try to apply to the AIDS centre. If they don't have a medical specialist I need, I go to the clinic but I don't inform them about my [HIV-] positive status. I feel better that way. (HIV-positive woman, Kiev, Ukraine)

### Broader community and societal discrimination

In our study, couples reported discrimination, ranging from excessive questioning about the word ‘HIV’ appearing on a participant's organisational letterhead, to rejection by insurance companies.

One of the Ukrainian participants reported having difficulty in securing a car loan because she worked for an HIV service organisation.

There was such a moment when I was buying a car. I was paying a rather good pre-payment – 25% of total cost and when I presented an income statement – it was okay, I have a decent salary – it had the name of my employer. I received a phone call from the bank asking a lot of questions about the organisation I work for. I think it was connected with that, and because of that I was asked to present a number of additional documents they didn't mention before, and find a guarantor, although with such big pre-payment the guarantor is not required. (HIV-positive woman, Kiev, Ukraine)

Some countries have policies barring entry by travellers who are HIV-positive. One HIV-positive woman interviewed in Ukraine reported that she had been refused a US travel visa because she was HIV-positive.

### Disclosure of HIV status

Seventy-three per cent of the South African participants and 90% of the Tanzanian participants had disclosed their discordant-couple status. Among the HIV-positive participants in South Africa and Tanzania, 81% had disclosed their sero-discordant status to someone, compared with 75% of the HIV-negative participants. Nonetheless, very few were living openly as an HIV-discordant couple. Those who were living openly often felt empowered by disclosing publicly and thought that there was some advantage in living openly as there was no need for people to probe or spread rumours:

I was comfortable talking to my family and friends, because I know they care about me. I had to explain my situation to them about my love for this girl. I told them I fell in love with her soul. I was actually safe because I knew her status. (HIV-negative man, Couple 4, South Africa)

Other couples who were living openly as a discordant couple wanted to be role models and to encourage greater openness about HIV.

I decided to share due to fear of AIDS-related illness. I also wanted to be a role-model, so that anyone who is infected can be open and go for treatment, rather than going to traditional healers. (HIV-positive woman, Couple 7, Tanzania)

We came out on a local educational TV series about our HIV-discordant status. We discuss our discordant-couple status with everybody and anybody. (HIV-positive man, same-sex Couple 23, South Africa)

The interviews revealed the complexity of *selective disclosure*, that is, individuals and couples appear to make conscious choices regarding the person(s) to whom they disclose. The study found that disclosure was limited to close family members, friends or to support groups (Table 1).

**Table 1.** Participants’ reported disclosure of HIV-discordant relationship in South Africa and Tanzania (percentages in parentheses).

Nature of disclosure	HIV-positive participants	HIV-negative participants	All African participants
No disclosure (i.e. only partner knows)	7 (19)	8 (25)	15 (22)
*Some* immediate family members of positive partner (e.g. sister, brother or parent) only	3 (8)	1 (3)	4 (6)
All immediate family members of positive partner (parent, sisters and brothers)	1 (3)	1 (3)	2 (3)
Some immediate family members of negative partner (e.g. sister, brother or parent) only	0 (0)	3 (9)	3 (4)
Immediate family members on both sides	2 (6)	3 (9)	5 (7)
Immediate family members and friends	19 (53)	13 (41)	32 (47)
Other (disclosed to friend, colleague, pastor/priest or support group members only)	4 (12)	3 (9)	7 (10)
Total	36	32	68

Notes: This information was obtained from individual qualitative interviews, and was not available for Ukraine. Only a summary report of the qualitative data was obtained from Ukraine.

The decision to disclose selectively was often linked to the need for psychosocial support, as can be understood from the following comments:

I shared with a group of people living with HIV and AIDS because they share the common objective and I receive counselling support from the group. (HIV-positive man, Couple 5, Tanzania)

In Ukraine, closest relatives such as parents were informed in some cases, but couples chose to keep their HIV-discordant status confidential.

Practically no one in my family knows about my problem. Only close friends and my partner, the person I live with, know. I don't hide it [HIV status] but I don't think one should yell about it. (HIV-positive woman, Kiev, Ukraine)

In the case of non-disclosure (22% of participants), the fear of stigma and discrimination was the overwhelming reason for non-disclosure, evidenced by the testimonies of several participants:

We made a decision with my partner to keep her HIV-positive status to ourselves because of the stigma and discrimination attached to the condition. (HIV-negative man, Couple 10, South Africa)

I have decided with my partner not to disclose my status as my mother is hypertensive and is easily disturbed by minor issues. Additionally, I do not want to disclose my status to my two brothers and sister as they all abuse alcohol, and after their drinking spree, they will abuse me verbally. (HIV-positive woman, Couple 13, South Africa)

I have not shared with anybody about being discordant, only with my partner. (HIV-positive man, Couple 2, Tanzania)

This couple from Tanzania (see the previous quotation) explained that the reasons for not disclosing their discordant relationship were that they thought that family members did not have knowledge of HIV and of HIV-discordant couples. They also feared stigma and discrimination. The couple lived in a rented house, and feared that they might be evicted from their house and become isolated and labelled if people found out about the partner's HIV-positive status. This was reiterated by another Tanzanian couple who said that they did not want to disclose to their family members and friends because they perceive them to have insufficient knowledge of HIV discordance, and the couple feared being stigmatised. The HIV-positive partner said that he did not want to disclose his status because he was still healthy, but said that he planned to disclose later when he became sick and bed-ridden.

One South African woman feared the consequences of disclosure to her mother, and to her HIV-negative partner's family, whom she considered to be very religious. She explained it as follows:

My mother would be shunned and discriminated against. I am also concerned about the church and what they would say … there are views of HIV as being promiscuous – the parents of my partner are old and very religious … I am worried how it would affect them. (HIV-positive woman, Couple 5, South Africa)

## Discussion

This study provides rich insight into the experiences of stigma and discrimination among HIV-discordant couples in South Africa, Tanzania and Ukraine. It is one of few studies focusing on couples and that explored the psychosocial dimensions of living in a HIV-discordant relationship, beyond an individual focus and/or a biomedical understanding of HIV discordance.

Disclosure of HIV status is generally thought to be an affirmative and empowering action that assists people in receiving support and understanding, and one that minimises psychological distress (Gillet & Parr 2010; Muhimbuura, Ssegujja, Ssali, Tumwine, Nekesa, Nannungi, *et al*. 2014; Sayles, Ryan, Silver, Sarkisian & Cunningham 2007). In contrast to other studies that focus on disclosure of HIV status to potential and current sex partners by HIV-positive people, our study focused on disclosure of the HIV-discordant relationship. The majority of South African (73%) and Tanzanian (90%) participants had disclosed their HIV-discordant couple status, but only selectively. This selective disclosure is not surprising, as decisions around disclosure are personal and influenced by many factors, including family dynamics, economic dependence, gender inequalities and power (International HIV/AIDS Alliance 2007). An Australian study found that disclosure may open up tensions, fears, scrutiny, unspoken concerns or unresponsiveness which tend to amplify feelings of difference rather than provide therapeutic release (Persson & Richards 2008). An American study found that HIV-positive people manage the anticipated identity challenges associated with status disclosure in several ways, including non-disclosure, timing the disclosure or selective disclosure to minimise the negative effects on identity (Frye *et al*. 2009). In both South Africa and Tanzania, the majority of the HIV-positive participants were women. Other studies have suggested that the disclosure of HIV infection is more likely to provoke stigma in patriarchal and sexist settings and that the negative consequences of HIV infection and disclosure are greater for women than for men (Castro & Farmer 2005; Nyblade, Pande, Mathur, MacQuarrie, Kidd, Banteyerga, *et al*. 2003; Talley & Bettencourt 2010). Selective disclosure was also found to be a coping mechanism in a multi-country study in Ethiopia, Tanzania and Zambia (Nyblade *et al*. 2003). It has been suggested that the emphasis on the ‘therapeutic value of disclosure obscures the complexities of HIV stigma as socially produced and lived’ (Persson & Richards 2008: 73).

Participants' reasons for non-disclosure related to fear of stigma and discrimination, evidenced by the poignant testimonies from various participants. In Ukraine, participants chose to keep their HIV status to themselves, and not make it a topic for discussion. Other studies have also shown that disclosure of HIV status is particularly difficult (Cloete *et al*. 2008; Persson & Richards 2008) and that high levels of perceived stigma limit disclosure of HIV status (Steward *et al*. 2008). A Ugandan study that explored couples’ explanations for discordance, challenges and prevention strategies found that couples feared disclosing their discordant relationship, in part due to a fear that others would not understand HIV discordance (Bunnell, Nassozi, Marum, Mubangizi, Malamba, Dillon, *et al*. 2005). A multi-country African study found that many participants feared that disclosure would lead to generalised stigma and result in more specific repercussions, such as blame for HIV infection and loss of family support (Nyblade *et al*. 2003). The emphasis on disclosure has been criticised and it has been argued that non-disclosure could be enabling, as it allows HIV-positive individuals a sense of ‘control’ over their lives and allows them to assume a ‘socially normative identity’ (Persson & Richards 2008).

The fear of stigma and discrimination expressed by participants was not unfounded. In our study, 60% of the Tanzanian participants and 21% of the South African participants had experienced some form of discrimination. Almost double the number of HIV-positive participants (43%) compared with 24% of the HIV-negative participants had experienced some form of discrimination, ranging from gossip, rumours, name-calling and ‘labelling’ to overt discrimination by family members and friends, and broader societal discrimination. In a few instances, strained relationships and blame for HIV infection surfaced in current relationships, as was shown in a Ugandan study that explored couples' explanations for discordance, challenges and prevention strategies (Bunnell *et al*. 2005).

In our study, stigma and discrimination by families and/or friends expressed itself through the pressure on the HIV-negative partner to leave the relationship, seemingly linked to the fear of HIV transmission to the HIV-negative partner. Although HIV transmission within a discordant relationship is a real possibility, the fear of HIV transmission is exacerbated by the general lack of knowledge about discordance. The findings in our study are supported by other studies that explored the experiences of HIV-positive individuals. A study that examined HIV stigma and discrimination among more than 600 individuals in South Africa, Tanzania, Thailand and Zimbabwe found that blame and gossip were common (Maman, Abler, Parker, Lane, Chirowodza, Ntogwisangu, *et al*. 2009). Factors found to contribute to HIV stigma included fear of transmission, fear of suffering and death, and the burden of caring for sick individuals (Maman *et al*. 2009). Similarly, a multi-country study in Ethiopia, Tanzania and Zambia found that insufficient and inaccurate HIV knowledge, together with fear of death and disease, perpetuate beliefs in casual transmission and lead to avoidance of people with HIV (Nyblade *et al*. 2003). A South African study on the development of an AIDS-Related Stigma Scale found an inverse correlation between high stigma scores and AIDS knowledge (Kalichman, Simbayi, Jooste, Toefy, Cain, Cherry, *et al*. 2005).

In Ukraine, some HIV-positive participants reported overt discrimination by health-care professionals, but this did not emerge as an issue in South Africa and Tanzania. Other studies have found that health-care workers are often the source of stigma or they could be stigmatised because they care for PLHIV (Greeff, Uys, Holzemer, Makoae, Dlamini, Kohi, *et al*. 2008; Holzemer & Uys 2004; Jha & Madison 2009; Li & Liang 2009; Varaz Diaz & Neilands 2009). A study conducted in China among more than 1000 health service providers found stigmatising attitudes, similar to perceived social norms in the general population (Li & Liang 2009). Participants who were younger or reported personal contact with PLHIV were more likely to report positive attitudes to PLHIV and a low level of discrimination intent at work (Li & Liang 2009). Stigma that emanates from health professionals is a serious concern as it can limit access to services (Varaz Diaz & Neilands 2009). A Nepalese study found that health professionals lacked knowledge and sensitivity in providing health care to PLHIV, and that stigma and marginalisation seem to interfere with doctors' and other health professionals’ decisions to treat persons who they perceive to be at high risk for HIV infection (Jha & Madison 2009).

The limitations of our study include the limited sample size, the inclusion of only three countries, and only three same-sex couples (all of whom were from South Africa). A further limitation was the recruitment of couples through health professionals and non-governmental organisations that provide services to HIV-positive individuals. Participants are likely to have had better access to and greater use of services, and to be better informed than discordant couples in the three study countries in general. The highly selective nature of our study participants limits the generalisability of our findings. Important perspectives may have been missed, given the heterogeneity of the HIV pandemic in the three countries. Information on sensitive topics was self-reported, thus there is likely to have been some social desirability bias in participants’ responses.

The strengths of our study include the use of mixed quantitative and qualitative methods, and conducting qualitative interviews with each partner separately as well as both partners together, thus exploring the nuances and contrasting perspectives among partners. A further strength was the restriction of eligibility to participate in the study to known discordant couples who were in long-term relationships, as participants had had time to work through the challenges of disclosure to sex partners. The study also provided the opportunity to gain an understanding of couples’ decisions around disclosure and their experiences of HIV stigma and discrimination, as opposed to those of individuals.

## Conclusion

The study findings underscore the need for a human rights approach in combatting stigma and discrimination; accelerating or enhancing existing anti-stigma programmes and strengthening existing health services.

Stigma and fear of discrimination can inhibit people from disclosing their status to families, employers or health-care providers, from accessing specialised health-care services or from taking appropriate steps towards positive health, dignity and well-being (GNP+ & UNAIDS 2009; Mawar, Sahay, Pandit & Mahajan 2005). Although implementation lags behind, stigma and discrimination reduction are now recognised as national and international funding, policy and programme priorities (SANAC 2011; UNAIDS & WHO 2013).

The study findings highlight a general lack of understanding about HIV-discordant relationships among family members and friends of these couples. Recent encouraging developments have included the introduction of the *People Living with HIV Stigma Index* to provide a tool to measure and detect changing trends in relation to stigma and discrimination experienced by PLHIV (IPPF, GNP+, ICW & UNAIDS 2008). Implementing the PLHIV Index nationally is one way to build an understanding of and commitment to reduce stigma and discrimination. Other efforts include social mobilisation programmes to accelerate anti-stigma programmes in the workplace, in faith-based settings, in communities and in the media to address stigmatising attitudes and discriminatory practices.

In general, the provision of couple-sensitive health services is under-developed, but a re-orientation of health services towards the needs of couples is both cost-effective and can overcome challenges of stigma, discrimination and disclosure (WHO 2014). As many HIV-positive people report considerable stigma and discrimination in health-care settings, training of health-care workers about HIV and HIV-related discrimination, and the establishment of codes of conduct, should help to overcome these problems (Holzemer & Uys 2004; International HIV/AIDS Alliance 2007; Jha & Madison 2009; WHO 2014).

HIV discordance should form an integral part of the global and national response to the HIV epidemic. National plans and programmes should be located within an overall human rights approach and should place greater emphasis on dealing with stigma and discrimination, which cannot be separated from the need for a supportive policy, programme and resource environment.
